# Long non-coding RNAs as the critical regulators of PI3K/AKT, TGF-β, and MAPK signaling pathways during breast tumor progression

**DOI:** 10.1186/s12967-023-04434-7

**Published:** 2023-08-18

**Authors:** Amirhosein Maharati, Meysam Moghbeli

**Affiliations:** 1 Student Research Committee, Faculty of Medicine, Mashhad University of Medical Sciences, Mashhad, Iran; 2 Department of Medical Genetics and Molecular Medicine, School of Medicine, Mashhad University of Medical Sciences, Mashhad, Iran

**Keywords:** Long-non coding RNAs, PI3K/AKT, MAPK, TGF-β, Cancer, Diagnosis

## Abstract

Breast cancer (BC) as one of the most common causes of human deaths among women, is always considered one of the global health challenges. Despite various advances in diagnostic and therapeutic methods, a significant percentage of BC patients have a poor prognosis due to the lack of therapeutic response. Therefore, investigating the molecular mechanisms involved in BC progression can improve the therapeutic and diagnostic strategies in these patients. Cytokine and growth factor-dependent signaling pathways play a key role during BC progression. In addition to cytokines and growth factors, long non-coding RNAs (lncRNAs) have also important roles in regulation of such signaling pathways. Therefore, in the present review we discussed the role of lncRNAs in regulation of PI3K/AKT, MAPK, and TGF-β signaling pathways in breast tumor cells. It has been shown that lncRNAs mainly have an oncogenic role through the promotion of these signaling pathways in BC. This review can be an effective step in introducing the lncRNAs inhibition as a probable therapeutic strategy to reduce tumor growth by suppression of PI3K/AKT, MAPK, and TGF-β signaling pathways in BC patients. In addition, considering the oncogenic role and increased levels of lncRNAs expressions in majority of the breast tumors, lncRNAs can be also considered as the reliable diagnostic markers in BC patients.

## Background

Breast cancer (BC) is the second-most common cause of cancer-related deaths that is considered as a health challenge among women [1]. Novel diagnostic and therapeutic strategies have decreased the mortality rate of breast cancer. However, approximately 279,100 new cases and 42,690 deaths due to breast cancer have occurred worldwide in 2020 [2]. Although, chemotherapy, radiotherapy, and surgery are major treatment options for the breast cancer, these modalities are not effective for the metastatic breast cancer patients [3, 4]. In fact, tumor metastasis is responsible for about 90% of cancer-related mortalities. Prevention of the tumor metastasis has been a major obstacle in the treatment of breast cancer. Therefore, investigating the underlying mechanisms of breast cancer metastasis is required to introduce novel and efficient therapeutic targets to reduce breast tumor invasion and metastasis. Early detection of breast cancer is critical for effective treatment. Diagnostic methods typically involve examination, ultrasonography, magnetic resonance imaging, mammography, and biopsy. Besides, constitutional treatments usually entail mastectomy, lumpectomy, hormone therapy, chemotherapy, and radiotherapy [5]. Non-coding RNAs (ncRNA) are considered as the key regulators of BC development and metastasis that can be suggested as the novel therapeutic approachs [6–8]. Long non-coding RNAs (lncRNAs) are a group of noncoding RNAs that are implicated in pivotal cellular processes in several tumors via interactions with protein, RNA, and DNA [9]. LncRNAs function as microRNA sponges in the cytoplasm and regulate gene expression post-transcriptionally through guiding RNA-binding proteins that are involved in active polysomes and mRNA decay [10–13]. In the nucleus, lncRNAs modulate RNA processing, transcription, and chromatin remodeling [14]. They are involved in various processes, including organogenesis, embryogenesis, and tumorigenesis [15, 16]. LncRNAs are implicated in the growth, metastasis, aggressiveness, migration, and programmed cell death of various cancers [17, 18]. Impared expression of lncRNAs has been detected in multiple human cancers, and they are pivotal regulators of tumor occurrence and progression via diverse signaling pathways [19, 20]. They are potent markers in early tumor diagnosis and targeted therapy [21–23]. Regarding the role of lncRNAs in regulation of signaling pathways in breast tumor cells, in the present review we discussed their role in regulation of PI3K/AKT, TGF-β, and MAPK signaling pathways during breast tumor progression and metastasis to suggest them as the reliable diagnostic and therapeutic options among BC patients (Table 1).

**Table 1:** Role of lncRNAs in regulation of PI3K/AKT, MAPK, and TGF-β signaling pathways in breast tumor cells

Study	Year	LncRNA	Target	Samples	Function	Clinical application
TU [32]	2022	LINC01133	PRR5/AKT	MDA-MB-231, MDA-MB-468, and Hs578T cell lines Xenograft model	Oncogene	Diagnosis
SADEGHALVAD [36]	2022	HOTAIR	PI3K/AKT	MCF-7 cell line	Oncogene	Diagnosis
LI [40]	2019	HOTAIR	PI3K/AKT	MCF-7 and SKBR3 cell lines	Oncogene	Diagnosis
HE [41]	2022	KB-1980E6.3	PI3K/AKT	51T 51N* BT-549, MDA-MB-231, SKBR3, MDA-MB-468 and MCF-7 cell lines Xenograft model	Oncogene	Diagnosis and prognosis
HAN [42]	2019	GHET1	c-Myc and PI3K/AKT pathways	30T 30N MCF-7 cell line Xenograft model	Oncogene	Diagnosis
NONG [46]	2021	FOXD2-AS1	PI3K/AKT	60T 60N MCF-7 cell line	Oncogene	Diagnosis
SHENG [47]	2020	SOX21-AS1	PI3K/AKT	88T 88N MCF-7, BT-20, MDA-MB-231, and MCF-10A cell lines Xenograft model	Oncogene	Diagnosis and prognosis
FANG [49]	2022	MBNL1-AS1	miR-423-5p/CREBZF	60T 60N MCF-10A, BT474, MDA-MB-231, MDA-MB-453, ZR-75–30, and MCF-7 cells lines Xenograft model	Tumor suppressor	Diagnosis and prognosis
ZHANG [51]	2020	ZFAS1	miR-589/PTEN	MCF-10A, T47D, MCF-7, MDA-MB-435 and BT-549 cell lines	Tumor suppressor	Diagnosis
GAO [52]	2019	PTENP1	miR-20a/PTEN	52T 52N MDA-MB-231, T-47D and MCF-7 cell lines Xenograft model	Tumor suppressor	Diagnosis and prognosis
SHI [55]	2018	PTENP1	miR-19/PTEN/PI3K/Akt	20T 20N MCF-7 and MDA-MB-231 cell lines	Tumor suppressor	Diagnosis
LEI [56]	2022	DUXAP8	PI3K/AKT and EZH2	50T 50N MCF-12A, MCF-12 F, MCF-7, T47D, ZR-75–1, HCC-1806, MDA-MB-468, BT-549, and MDA-MB-231 cell lines Xenograft model	Oncogene	Diagnosis and prognosis
WANG [59]	2021	SNHG6	miR-543/LAMC1	28T 28N MCF-7, SK-BR-3, MDAMB-231, and BT-549 cell lines Xenograft model	Oncogene	Diagnosis
LI [60]	2019	UCA1	EZH2 and AKT	10 ER + 10 ER- MCF-7, T47D, LCC2, and LCC9 cell lines	Oncogene	Diagnosis
FANG [61]	2022	TTN-AS1	miR-107/ZNRF2	MCF-7 cell line	Oncogene	Diagnosis
ZHOU [74]	2022	HULC	IGF1R	MCF7 and MDA-MB-231 cell lines Xenograft model	Oncogene	Diagnosis
ZHANG [75]	2021	IGF2-AS	IGF2	95T 95N MCF-7, SK-BR-3, T47D, and MDA-MB-231 cell lines Xenograft model	Tumor suppressor	Diagnosis and prognosis
LI [76]	2018	GAS5	miRNA-196a-5p/FOXO1	103T 50N MDA-MB-231 and MDA-MB-468 cell lines	Tumor suppressor	Diagnosis and prognosis
ZHANG [77]	2019	ZEB2‐AS1	ZEB2	98T 98N MCF‐10A, T47D, MDA‐MB‐435, MCF‐7, and MDA‐MB‐23 cell lines Xenograft model	Oncogene	Diagnosis and prognosis
LIN [81]	2020	BDNF-AS	RNH1/TRIM21	162T 162N MCF-7R and MDA-MB-231 cell lines Xenograft model	Oncogene	Diagnosis and prognosis
CHEN [84]	2019	HOTAIR	PTEN	SK-BR-3 cell line Xenograft model	Oncogene	Diagnosis
CHEN [88]	2020	Linc00839	PI3K/AKT	837T 105N (TCGA) 32T 32N MCF‐7, BT549 and MDA‐MB‐231 cell lines Xenograft model	Oncogene	Diagnosis and prognosis
ZHONG [90]	2021	AC012213.3	RAD54B	1109T 113N (TCGA) 11T 11N MCF-7, T47D, MDA-MB-231 and MDA-MB-469 cell lines	Oncogene	Diagnosis and prognosis
TAO [92]	2020	SCAMP1-TV2	PUM2	20T 20N MCF-10A, MCF-7, and MDA-MB-231 cell lines Xenograft model	Oncogene	Diagnosis
WANG [100]	2021	ARHGAP5-AS1	SMAD7	1109T 113N (TCGA) MDA-MB-231 and LM2 cells, SKBR3 and BT549 cell lines	Tumor suppressor	Diagnosis
WU [101]	2017	CCAT2	TGF-β	60T 60N LCC9, MDA-MB_231 and MCF-7 cell lines	Oncogene	Diagnosis
HOU [102]	2018	Linc-ROR	TGF-β	94T 94N MDA-MB-231 and MCF-7 cell lines Xenograft model	Oncogene	Diagnosis and prognosis
LI [103]	2021	HNF1A-AS1	miR-363/SERTAD3	82T 82N MCF-7, BT549, ZR-75–30, MDA-MB-231, HCC1937 and MDA-MB-436 cell lines Xenograft model	Oncogene	Diagnosis and prognosis
DONG [105]	2021	LINC00052	miR-145-5p/TGFBR2	45T 45N MDA-MB-231, MDA-MB-468, T47D, SKBR3 and MCF-7 cell lines	Oncogene	Diagnosis
ZHANG [106]	2019	CASC2	TGF-β	52T 52N LCC9, MDA-MB-231, and MCF-7 cell lines	Tumor suppressor	Diagnosis
LI [118]	2017	ANCR	RUNX2	25T 25N MCF7, T47D, MDA-MB-231, MDA-MB-231HM and BT549 cell lines Xenograft model	Tumor suppressor	Diagnosis
LI [119]	2021	TPA	TGF-β	MCF-7 cell line Xenograft model	Oncogene	Diagnosis
ZHOU [121]	2019	lncRNA-NORAD	TGF-β	21T 10N MDA-MB231 and MCF-7 cell lines Xenograft model	Oncogene	Diagnosis and prognosis
LI [127]	2018	AC026904.1 and UCA1	Slug	60T 60N MDA-MB-231 and luc-D3H2LN cell lines Xenograft model	Oncogene	Diagnosis and prognosis
REN [135]	2018	HOTAIR	H3K27	39T(invasive) 20T (in situ) MDA-MB-231 and MCF-7 cell lines Xenograft model	Oncogene	Diagnosis and prognosis
TANG [138]	2020	DCST1-AS1	ANXA1	MDA-MB-231, BT-549, T-47D, and MCF7 cell lines	Oncogene	Diagnosis
FANG [140]	2017	HOXA-AS2	miR-520c-3p/ TGFBR2/RELA	38T 38N MDA-MB-231, MDA-MB-453, and MCF-7 cell lines Xenograft model	Oncogene	Diagnosis and prognosis
NI [146]	2021	ADAMTS9-AS2	RPL22	62T 62N MDA-MB-231 and HCC1937 cell lines Xenograft model	Tumor suppressor	Diagnosis and prognosis
ZHOU [152]	2022	TGFB2-AS1	SMARCA4	281T 281N MDA-MB-231, SUM159PT and BT-549 cell lines Xenograft model	Tumor suppressor	Diagnosis and prognosis
ZHANG [161]	2021	CBR3-AS1	miR-25-3p/MEK4/JNK1	96T 96N MCF-7, T47D, MDA-MB-231 and HEK-293 T cell lines Xenograft model	Oncogene	Diagnosis and prognosis
CHEN [162]	2017	PTENP1	AKT and MAPK	MCF7 and 293T cell lines	Tumor suppressor	Diagnosis
LU [163]	2018	lncCAMTA1	miR-20b/VEGF	MDA-MB-231 cell line	Oncogene	Diagnosis
OUYANG [164]	2021	PRNCR1	miR-377/CCND2	64T 64N MDA-MB-231, MCF-7, BT-549, MDAMB-468 and SK-BR-3 cell lines	Oncogene	Diagnosis and prognosis
PENG [167]	2017	Linc- ROR	DUSP7	MCF-7 cell line	Oncogene	Diagnosis
CHEN [172]	2020	MIR100HG	miR-5590-3p/OTX1	20T 20N MDA-MB-231, MDA-MB-453, MDA-MB-468 and MDA-MB-415 cell lines Xenograft model	Oncogene	Diagnosis
ZENG [184]	2022	SNHG5	IGF2BP2	30T 30N Xenograft model	Oncogene	Diagnosis

*Tumor (T) tissues and Normal (N) margins

### PI3K/AKT signaling pathway

**Fig. 1:**
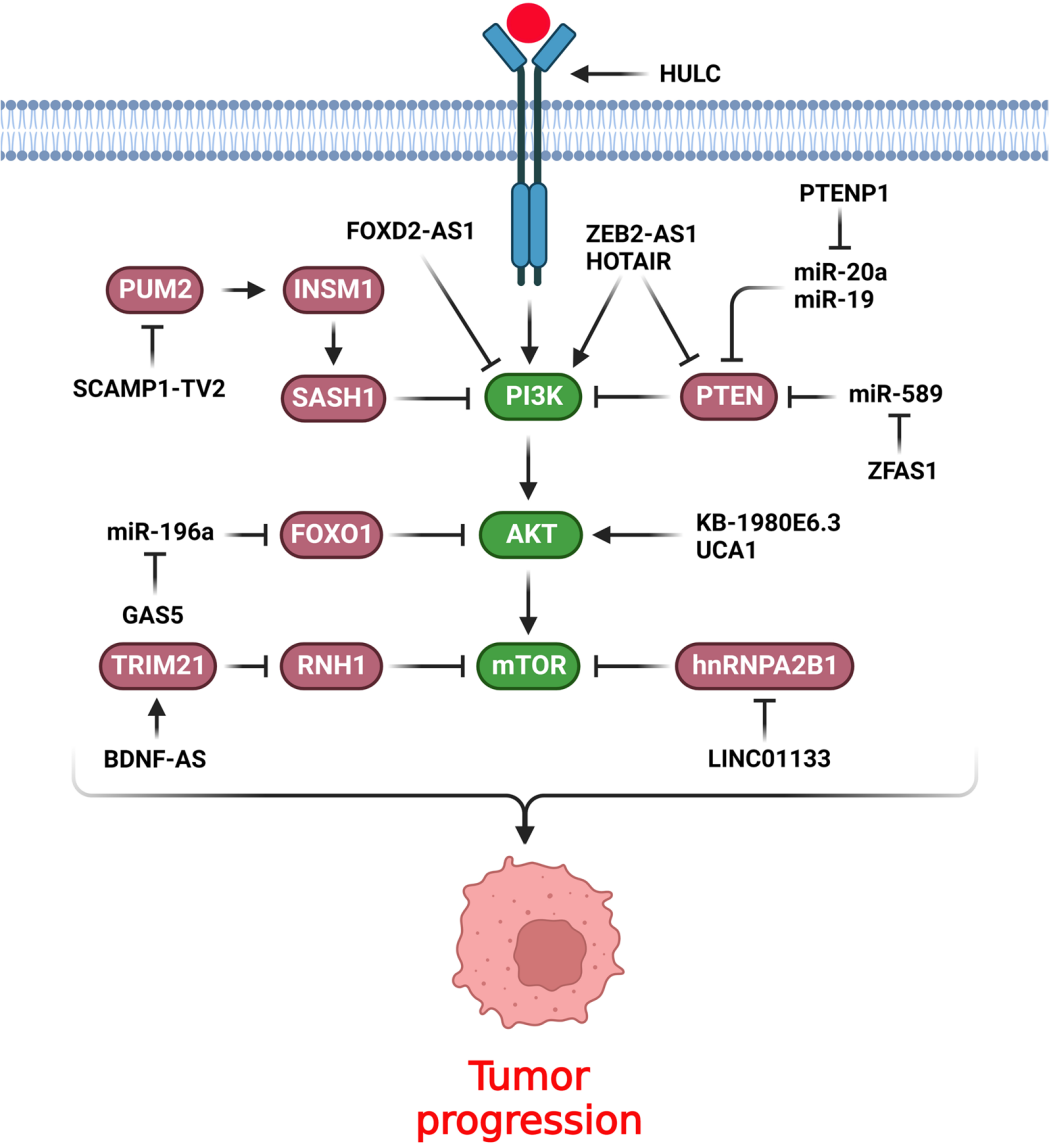
Role of lncRNAs during breast tumor progression by regulation of PI3K/AKT signaling pathway. (Created with BioRender.com)

PI3K/AKT/mTOR pathway is a crucial axis in regulation of various pathophysiological cellular processes such as cell proliferation, metabolism, and tumor progression [24, 25]. Majority of the growth factors, cytokines, and mitogens affects the cellular growth via the PI3K/AKT pathway. This signaling pathway is activated via various receptors such as receptor tyrosine kinases (RTKs), cytokine receptors, and G-protein-coupled receptors (GPCRs) that promote PI3K to produce PIP3. Subsequently, PIP3 activates the AKT to regulate cellular metabolism and growth via the modulation of various effectors such as GSK3β and mTOR. Despite the extracellular stimuli, AKT can also be activated by the other signaling pathways including WNT and TGF-β [25]. PI3K/Akt pathway functions as an oncogenic signaling axis in the progression of various cancers [26, 27]. PI3K hyper activation is critical in the pathogenesis of breast cancer that modulates cell survival, motility, growth, and metabolism [28, 29]. PI3K/Akt/mTOR pathway has a key role in endocrine resistance of breast tumor cells. Therefore, inhibitors of this pathway can be used in combination with other therapeutic modalities in breast cancer. It has been reported that PIK3CA mutations increased sensitivity toward the PI3K inhibitors [30]. LncRNAs have key roles during breast tumor progression by regulation of PI3K/AKT signaling pathway (Fig. 1). hnRNPA2B1 is an essential modulator of several normal processes such as mRNA stability and translation, RNA trafficking, and mRNA splicing [31]. LINC01133 increased cell proliferation in TNBC through PI3K-independent activation of AKT. LINC01133 stimulated PROTOR1 as a part of the mTORC2 complex via hnRNPA2B1 sponging that induced AKT [32]. The stimulated mTOR signaling pathway has been correlated with poor prognosis and reduced survival in BC patients [33]. Interactions between Polycomb Repressive Complex 2 (PRC2) and HOTAIR change the chromatin structure to accelerate the tumor cells metastasis [34, 35]. HOTAIR inhibition significantly down regulated the mTOR, AKT, and PI3K. The precise mechanism by which HOTAIR triggers the expression of PI3K, Akt, and mTOR is not fully understood; however, it is hypothesized that HOTAIR regulates transcription factors [36]. Doxorubicin (DOX) is one of the well-known and most effective drugs in BC; however, tumor cells may develop drug resistance that results in treatment failure [37–39]. HOTAIR inhibition reduced DOX resistance in BC cells via suppression of the PI3K/AKT/mTOR pathway following the negative regulation of PI3K, AKT and mTOR. HOTAIR inhibition also induced apoptosis in DOXR-MCF-7 cells via regulating Bax, Bcl-2, and caspase-3 [40]. KB-1980E6.3 up regulation was significantly associated with breast tumor progression and poor prognosis. KB-1980E6.3 increased the growth, migration, and aggressiveness of BC cells. KB-1980E6.3 was also significantly correlated with MMP-2, MMP-9, and vimentin. Moreover, it stimulated the PI3K/AKT pathway through AKT and PI3K phosphorylations [41]. GHET1 inhibition mitigated cell growth and invasion while promoted programmed cell death. GHET1 downregulation inhibited the c-Myc, which resulted in PI3K/AKT inactivation. Therefore, its inhibition reduced BC progression through PI3K/AKT repression. GHET1 inhibition also hindered the MCF-7 cell migration via negative regulation of MMP-2 and MMP-9. Accordingly, inhibition of GHET1 attenuated PI3K/AKT, c-Myc, and their downstream effectors such as MMP-2/9 and CCND1 [42]. FOXD2-AS1 has been found to be upregulated in bladder, gastric, and ovarian cancers and was also involved in the tumor cells migration, growth, invasion, and prognosis [43–45]. There was significant FOXD2-AS1 upregulation in antiadriamycin-resistant breast tumor cells and tissues. FOXD2-AS1 also down regulated the p-AKT and pPI3K to inhibit the PI3K/AKT signaling pathway in BC cells. Furthermore, inhibition of FOXD2-AS1 negatively regulated the growth, aggressiveness, and migration of BC cells while induced apoptosis and chemosensitivity [46]. There was SOX21-AS1 up regulation in BC tissues that was correlated with tumor stage, grade, metastasis, and clinical outcomes. SOX21-AS1 suppression significantly reduced EMT, cell growth, and invasion in BC cells. Repression of SOX21-AS1 also inhibited the PI3K/AKT signaling pathway, which underscored their potential interaction. Hence, SOX21-AS1 increased cell proliferation, invasion, and EMT via targeting the PI3K/AKT pathway in BC [47]. CREBZF belongs to the ATF/CREB family of transcription factors that regulates p53 mediated apoptosis [48]. The MBNL1-AS1 functioned as a tumor suppressor in BC through miR-423-5p/CREBZF axis that regulated PI3K/AKT pathway [49].

PTEN as a well-known tumor suppressor inhibits the PI3K/AKT axis via dephosphorylation of PIP3 [50]. ZFAS1 enhanced apoptosis while mitigated cell growth and migration via miR-589 sponging that up regulated PTEN to inhibit the PI3K/AKT pathway [51]. There were PTENP1 and PTEN downregulations in BC tissues in comparison with normal margins that was correlated with a higher TNM stage and decreased overall survival in BC patients. PTENP1 inhibited tumor proliferation, colony formation, invasion, and tumor growth in BC. It regulated the chemoresistance, apoptosis, metastasis, and proliferation of BC cells by regulation of miR-20a/PTEN axis that inhibited PI3K/Akt pathway [52]. DUXAP8 exerts an oncogenic function in various cancers via targeting EZH2 and PTEN [53, 54]. PTENP1 suppressed BC cell invasion, colony formation, and survival while induced cell death via miR-19 sponging and subsequent regulation of PTEN/PI3K/Akt axis [55]. DUXAP8 induced the radioresistance of BC cells via stimulation of the PI3K/AKT/mTOR pathway and inhibition of EZH2 target genes including RHOB and E-cadherin [56]. Laminin subunit gamma 1 (LAMC1) is an extracellular matrix protein that has key roles in basement membranes, cell proliferation, movement, and development [57]. It also enhances the development of hepatocellular carcinoma via the PTEN/AKT axis [58]. SHNG6 induced the breast tumor cell growth and movement via miR-543 sponging and subsequent activation of LAMC1/PI3K/AKT axis [59].

Tamoxifen (TAM) is a widely used endocrine therapy that operates as an antagonist of estrogen in BC patients [5, 6]. Although, tamoxifen therapy is effective for most of the ER+ breast cancers, many patients eventually develop resistance to tamoxifen [7, 8]. Therefore, understanding the underlying mechanism of TAM resistance will decrease its adverse effects and facilitate overcoming resistance and sensitizing breast tumors. Enhancer of zeste homolog 2 (EZH2) is a member of polycomb proteins that is involved in the modulation of tumorigenesis [28, 29]. There was remarkable UCA1 upregulation in tamoxifen-resistant breast cancer relative to sensitive samples. Loss of UCA1 in LCC2 cells interrupted the cell cycle at G2/M phase and dysregulated p21 and CCND1. EZH2 negatively regulated p21 transcription via H3K27me3, which was induced by UCA1 in BC cells. Inhibition of UCA1 significantly reduced CREB and p-CREB expression levels. There was a positive association between the expression levels of AKT and UCA1. Therefore, UCA1 modulated the CREB via targeting the AKT and PI3K/AKT signaling pathways [60]. TTN-AS1 induced the breast tumor cell proliferation, aggressiveness, and tamoxifen resistance by targeting miR-107/ZNRF2 axis. ZNRF2 inhibition activated the PI3K/AKT pathway, which resulted in increased tamoxifen resistance in BC cells [61].

Triple Negative Breast Cancer (TNBC) as the most aggressive form of BC is characterized by a lack of estrogen receptor (ER), epidermal growth factor receptor 2 (HER-2), and progesterone receptor (PR) that results in the failure of targeted therapies in these patients [62]. About two-thirds of TNBC patients have a poor chemotherapy response [63]. IGF binds with IGF1R receptor to activate anti-apoptotic pathways and cell growth via the PI3K/AKT and RAS/MAPK signaling axes [64]. The downstream pathways of IGF1R induce the stemness and epithelial-to-mesenchymal transition (EMT) [65]. IGF1R is associated with the expression of EMT-related markers (Twist and Snail) and self-renewal factors (SOX2, OCT4, and NANOG) [66–72]. Additionally, IGF1R promotes cell growth and inhibits apoptosis via the Ras/Raf/MEK, PI3K/AKT/mTOR pathways [64, 73]. The HULC-IGF1R axis increased the growth and metastasis of breast tumor cells. HULC also enhanced IGF1R transcription through acetylation of H3K9 histone, intergenic chromosomal loop construction, and interaction with cis-acting elements. Accordingly, the HULC-IGF1R axis increased cisplatin resistance by up regulation of stem cell markers [74]. There was significant downregulation of IGF2-AS in BC plasma, tissues, and cell lines. IGF2-AS down regulated the IGF2 via DNMT1 that inhibited PI3K/AKT/mTOR and tumor progression in BC [75]. There was GAS5 downregulation in TNBC tissues that was associated with an invasive morphology in TNBC patients. GAS5 impeded cell growth while enhanced apoptosis in TNBC cells. GAS5 also attenuated TNBC development via miR-196a-5p sponging, which subsequently overexpressed FOXO1 and inhibited PI3K/AKT phosphorylation [76]. ZEB2-AS1 induced the growth, metastasis, and EMT in TNBC cells through regulating the PI3K/Akt/GSK3β/Zeb2 axis and polymerization of F‐actin [77].

Hormone receptor (HR)-positive BC patients may develop intrinsic and acquired resistance to hormone therapies, which is responsible for tumor relapses in these patients [78]. PI3K or mTOR inhibitors are used to restrain drug resistance, underscoring the critical role of PI3K/AKT/mTOR in endocrine therapy resistance [79, 80]. BDNF-AS stimulated the mTOR signaling pathway in endocrine-resistant BCs and TNBCs by RNH1 protein degradation. MEF2A is a transcription factor that up regulates the BDNF-AS. Interaction between RNH1 and RISC was involved in the destruction of mTOR mRNA. TRIM21 is an E3 ligase that degraded RNH1. BDNF-AS functioned as molecular glue for the TRIM21/RNH1 interaction that resulted in RNH1 degradation [81]. Trastuzumab as an anti-HER2 monoclonal antibody deactivates the downstream pathways and inhibits the production of HER2 dimers, which results in the suppression of tumor growth while apoptosis induction [82]. Trastuzumab resistance is one of the leading causes of treatment failure in HER2-positive BC patients. Therefore, developing new targeted therapies to overcome chemoresistance is an ideal strategy that improves the survival rate of HER2-positive BC patients [83]. HOTAIR mediated PTEN methylation activated PI3K/AKT to promote trastuzumab resistance. HOTAIR inhibition downregulated CCND1, p-MAPK, and p-AKT while upregulated P27 and PTEN. The upregulation of TGF-β, Snail, and Vimentin and impaired expression of CDH1 were reported in resistant BC cells. HOTAIR regulated acquired resistance through epigenetic changes, including demethylation of TGF-β and PTEN methylation, which increased the HER2-independent MEK/MAPK activity and subsequent proliferation and invasion of malignant cells [84].

Myc oncogene is a pivotal transcription factor that is implicated in tumor cell reprogramming and apoptosis [85, 86]. Lin28B facilitates the Myc nuclear translocation to increase AKT phosphorylation [87]. There was remarkable upregulation of Linc00839 in BC tissues that was corelated with an unfavorable prognosis. Linc00839 was regulated by Myc, which in turn modulated the expression of Lin28B and Myc proteins. Moreover, Linc00839 induced chemoresistance and growth via the PI3K/AKT signaling pathway and also phosphorylated the P38, STAT3, and Akt proteins. Therefore, Linc00839 ameliorated the breast tumor cell proliferation and chemoresistance through the Lin28B-induced Myc upregulation and PI3K/AKT activation [88]. RAD54B exhibits oncogenic function due to its pivotal role in the DNA repair and genomic instability [89]. AC012213.3 inhibition remarkebaly restrained the growth and invasion of BC cells. AC012213.3 regulated the RAD54B/PI3K/AKT axis to exert its oncogenic function in tumor cells. There was also AC012213.3 upregulation in BC tissues that was correlated with survival. Moreover, AC012213.3 targeted RAD54B, which resulted in tumor progression [90]. RNA-binding proteins (RBPs) are the key posttranscriptional regulators that have pivotal roles in tumor progression. Pumilio RNA binding family member 2 (PUM2) is a PUF family member of RBPs that modulates malignant tumors [91]. SCAMP1-TV2 suppression impeded the malignant characteristics of BC cells via decreasing their attachment to PUM2 and inducing the binding of PUM2 to INSM1 which finally downregulated the INSM1. Reduced expression of INSM1 inhibited SASH1 which suppressed the PI3K/AKT pathway in breast tumor cells [92].

### TGF-β signaling pathway

**Fig. 2:**
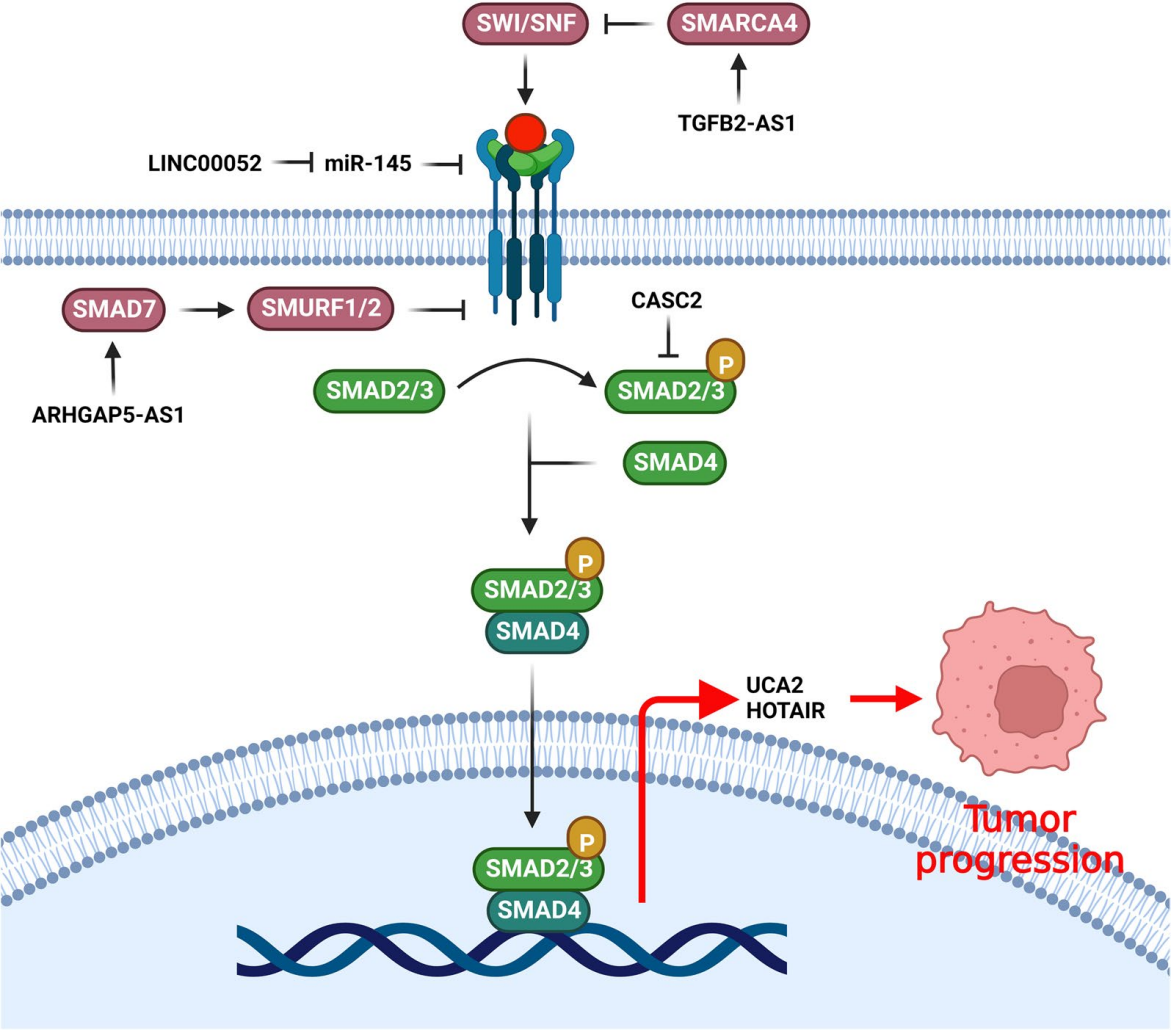
Role of lncRNAs during breast tumor progression by regulation of TGF-β signaling pathway. (Created with BioRender.com)

Transforming growth factor β (TGF-β) signaling is an important pathway during the development of different tumors. Deregulation of TGF-β pathway facilitates tumor cell proliferation, dissemination, metastasis, and immune scape [93]. TGF-β ligand binds to the TGF-βII/I receptors that phosphorylates and activates the SMAD2/3 (R-SMADs). Activated R-SMADs form a complex with SMAD4 and translocates to the nucleus to regulate the transcription of TGF-β target genes [94]. TGF-β has key role during breast cancer metastasis. Regarding the role of TGF-β in regulation of EMT process and stemness, it has a pivotal role in modulation of breast cancer stem cells [95]. LncRNAs have pivotal roles during breast tumor progression by regulation of TGF-β signaling pathway (Fig. 2). SMAD6 and SMAD7 inactivate TGF-β pathway via antagonistic signals and feedback loops [96]. SMAD7 is an important modulator of TGF-β signaling that inhibits the pathway through several proceses [97]. It functions within the cytoplasm by interfering with SMAD2/3 for the binding site of TGFβR1, thereby preventing the SMAD2/3 phosphorylation and suppressing signal transduction [98]. SMAD7 also facilitates the recruitment of SMURF1 and SMURF2 to TGF-βR1 and subsequent receptor degradation [99]. ARHGAP5-AS1 inhibited breast tumor cell migration via stabilizing SMAD7 [100]. CCAT2 suppression restrained tumor growth and invasion while induced apoptosis in BC cells via targeting the TGF-β signaling pathway. CCAT2 downregulation negatively regulated the TGF-β, α-SMA, and Smad2 proteins in BC cells [101]. There were linc-ROR upregulations in BC cell lines and tissues that was associated with an unfavorable prognosis. Linc-ROR also increased in-vivo tumor growth and invasion in BC via up regulation of the key components in TGF-β pathway [102]. HNF1A-AS1 induced TAM resistance in BC cells via the miR-363/SERTAD3 axis that promoted TGF-β/Smad [103]. TGFBR2 and TGF-β combination stimulates the TGF-β/Smad pathway that results in p21 and p15 up regulations, while c-Myc down regulation [104]. There was LINC00052 upregulation in BC that sponged miR-145-5p to induce TGF-βR2 expression [105]. CASC2 reduced BC progression by down regulation of TGF-β, Smad2, and a-SMA [106].

Epithelial-mesenchymal transition (EMT) process attenuates cell–cell adhesion via downregulation of the epithelial markers such as E-cadherin, whereas it induces cell mobility and the expression of mesenchymal markers including fibronectin, CDH2, and vimentin. EMT has also a pivotal role in breast tumor cell invasion and metastasis [107, 108]. TGF-β induces and preserves EMT process to promote tumor metastasis [109–111]. Runt-related transcription factor 2 (RUNX2) is a well-known regulator of osteoblast morphology and osteogenesis. The role of RUNX2 in tumorigenesis has been also investigated in recent years [112–117]. RUNX2 is involved in TGF-β-mediated EMT in which it is up regulated by TGF-β1 [115]. TGF-β1 inhibited the acetylation of the ANCR promoter and promoted HDAC3 enrichment at the ANCR promoter. ANCR modulated the TGF-β pathway via negative regulation of RUNX2. TGF-β also down regulated the ANCR that reduced BC cell invasion and migration. ANCR mitigated TGF-β1-induced EMT by RUNX2 down regulation [118]. LncRNA TPA provoked the aggressiveness and metastasis of BC through the initiation of EMT by targeting TGF-β signaling pathway [119]. RUNX2 provokes the EMT process by downregulation of CDH1 while up regulation of Vimentin and Snail2. TGF-β promotes BC metastasis by RUNX2 upregulation [120]. There was lncRNA-NORAD upregulation in BC, which was correlated with tumor growth, invasion, and poor prognosis. LncRNA-NORAD modulated TGF-β pathway to up regulate the RUNX2 [121]. Several pleiotropic transcription factors, such as Twist, ZEB1/2, Slug, and Snail, regulate the EMT process by promoting the mesenchymal while suppressing epithelial markers. Multiple intracellular and extracellular pathways also regulate the expression of these critical transcription factors [122]. TGF-β signaling pathway regulates a broad range of downstream genes, such as EMT transcription factors, via Smad and non-Smad pathways [123, 124]. TGF-β regulates the interaction between Smad and ERK signaling pathways, which enhance and preserve the expression of Slug [125, 126]. There were significant upregulations of UCA1.1 and AC026904 by TGF-β pathways. UCA1.1 and AC026904 enhanced the expression of Slug, thereby inducing EMT process and tumor metastasis. AC026904.1 played as a ceRNA to stimulate SLUG, whereas UCA1 up regulated the Slug via miR-203a and miR-1 spongings [127]. TGF-β promoted the DOX resistance and EMT process by UCA1 up regulation in breast tumor cells [128].

Cancer-associated fibroblasts (CAFs) as the pivotal components of the tumor microenvironment are involved in tumor growth, angiogenesis, invasion, and chemoresistance through cytokines and growth factors such as PDGF, b-FGF, VEGF, and TGF-β1 [129–133]. CDK5 has been reported to be a critical regulator of TGF-β1-mediated EMT during breast cancer progression [134]. CAFs increased the metastasis of BC cells via TGF-β1, which regulates the stroma-tumor cell interaction. CAFs also activated HOTAIR to promote EMT. HOTAIR was a direct transcriptional target of SMAD2/3/4. CAFs induced HOTAIR transcription to promote H3K27 trimethylation of EGR-1 and CDK5RAP1 promoters that up regulated the CDK5 and increased EMT. CAFs also activated HOTAIR, which in turn reinforced the EMT process. SMAD2/3/4 directly regulated the HOTAIR transcription. CAFs induced EMT and metastasis in BC cells via regulation of TGF-β1-mediated interactions between cancer cells and stromal cells [135]. Annexin A1 (ANXA1) belongs to the Ca2+ -dependent phospholipid-binding protein family that is involved in regulation of the leukocytes mediated immune responses [136]. ANXA1 is also engaged in regulation of signaling pathways to affect the tumor cell growth, invasion, angiogenesis, and apoptosis [137]. DCST1-AS1 attached to ANXA1 to promote TGF-β-mediated EMT in BC cells. DCST1-AS1 also increased the paclitaxel and doxorubicin resistances of BT-549 cells through targeting ANXA1. Additionally, DCST1-AS1 inhibition affected TGF-β-induced MMP2/9 releasing in MDA-MD-231 cells. DCST1-AS1 regulated the IGF2BP1; thereby DCST1-AS1 may perform its regulatory function on ANXA1 mRNA via targeting IGF2BP1. DCST1-AS1 enhanced TGF-β-mediated EMT and induced resistance to paclitaxel and doxorubicin via regulation of ANXA1 in TNBC cells [138].

RELA (p65, NF-κB3) is a NF-κB family member that modulates the proliferation and malignancy of several tumors via pro-survival and pro-inflammatory factors [139]. There was HOXA-AS2 upregulation in BC that was correlated with invasion, lumph node involvement, TNM staging, and survival. HOXA-AS2 inhibition significantly suppressed breast tumor cell growth via targeting miR-520c-3p/RELA and TGFBR2 axis [140]. RPs are a type of RNA-binding protein that are found in all cells [141]. RPL22 is a 60S ribosomal subunit and is associated with bacterial macrolide resistance by its mutation [142]. RPL22 promotes TGF-β pathway during tumor progression [143–145]. There was significant reduced expression of ADAMTS9-AS2 in TNBC samples compared to normal tissues that was correlated with tumor size, lymph node involvement, a higher TNM stage, patient age, and worse prognosis. ADAMTS9-AS2 attenuated the growth and invasion of TNBC cells via the TGF-β-mediated regulation of the ADAMTS9-AS2/RPL22 axis [146]. LncRNAs interact with PRC, SWI/SNF, and Pol II machinery to regulate gene expression inside the nucleus [34, 147–150]. Chromatin remodeling plays a crucial role in regulation of gene expression via proteins or protein complexes. SWI/SNF complex as a chromatin remodeller moves the nucleosomes and makes the DNA more accessible via recruitment of transcription factors to certain DNA regions [151]. There was TGFB2-AS1 downregulation in the metastatic TNBC patients. TGFB2-AS1/SMARCA4 interaction suppressed the SWI/SNF that was followed by TGFB2 and SOX2 down regulations. There was also up regulation of mesenchymal markers (slug, vimentin, and fibronection) while downregulation of epithelial markers (β-catenin) in breast tumor cells [152].

### MAPK signaling pathway

**Fig. 3:**
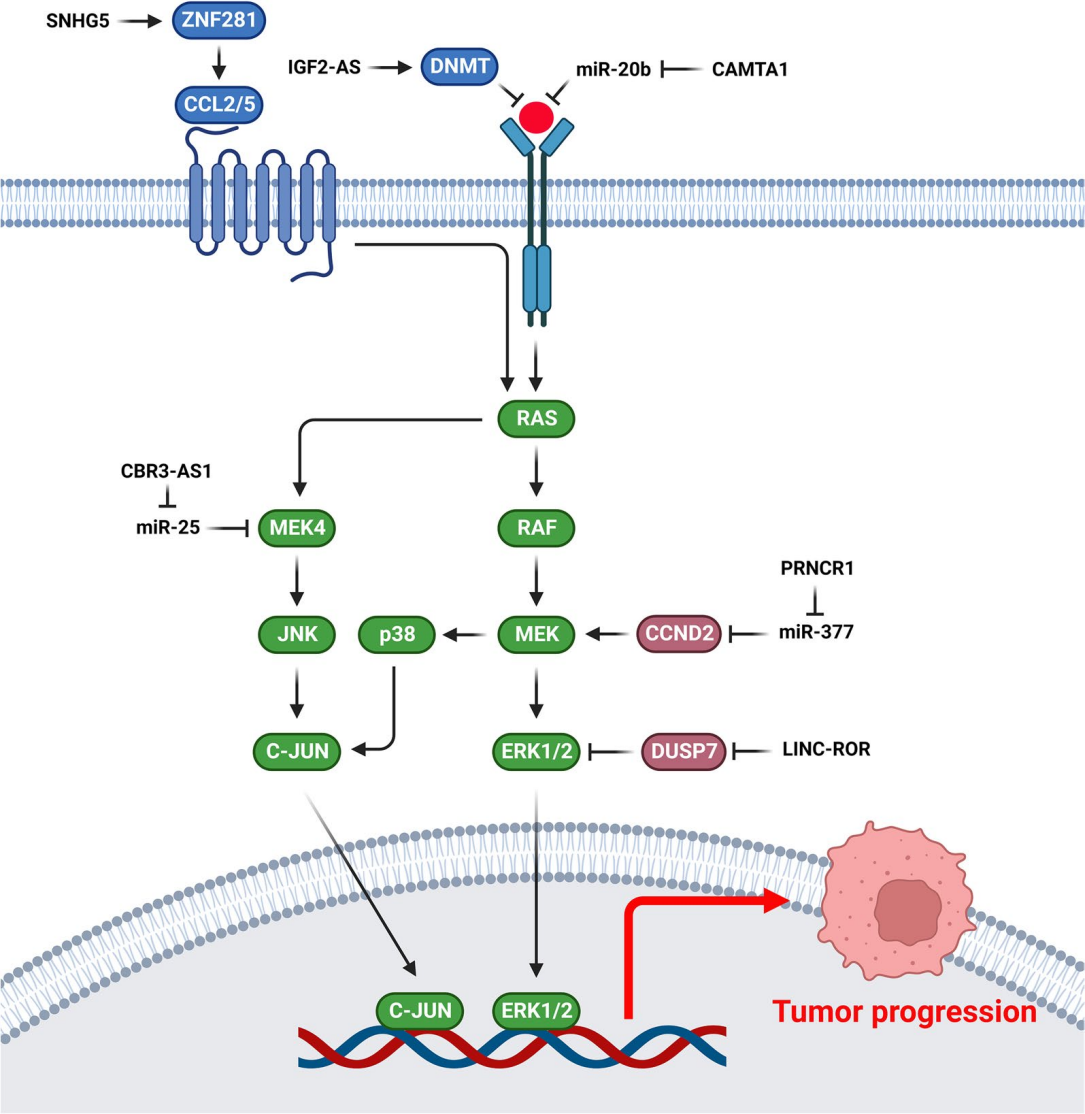
Role of lncRNAs during breast tumor progression by regulation of MAPK signaling pathway. (Created with BioRender.com)

MAPK is a pivotal pathway for the regulation of tumor invasion in multiple malignant cancers [153–156]. The genes involved in the MAPK pathway play important roles in numerous biological processes, including apoptosis, proliferation, and differentiation [157, 158]. MAPK cascades transmit and amplify the extracellular stimuli such as growth factors and steroid hormones which are associated with cell proliferation and apoptosis. ERK is the most important effector of the MAPK during breast cancer progression. Steroid hormones can activate MAPK. It has been reported that MAPK activation was higher in about half of breast tumors compared with normal margins [159]. LncRNAs have key roles during breast tumor progression by regulation of MAPK signaling pathway (Fig. 3). MEK4 activates JNK via phosphorylation that promotes its nuclear accumulation to activate the ELK21, ATF-22, and c-Jun transcription factors [160]. CBR3-AS1 upregulation was correlated with poor prognosis in ADR-resistant BC cell lines and patients. CBR3-AS1 increased resistance to ADR in BC cells through targeting the miR-25-3p/MEK4/JNK1 axis and intensifying the MAPK pathway [161]. PTENP1 suppressed the migration and growth of BC cells via the AKT signaling pathway and cell cycle associated genes such as CDK2 and cyclin A2. PTENP1 also inhibited the growth and migration of BC cells via the MAPK signaling pathway by repressing the phosphorylation of key proteins in this pathway including Erk1/2 and p38 [162]. LncCAMTA1 promoted the BC progression via miR-20b/VEGF axis that induced JAK/STAT3 and MAPK/ERK pathways [163]. PRNCR1 inhibition impaired cell survival and increased apoptosis and the number of cells in the G0/G1 phase following increased Bax and decreased Bcl-2 expressions. PRNCR1 inhibition reduced CCND2 expression, while suppression of miR-377 restored CCND2 expression levels. CCND2 also increased p38 MAPK and MEK1 phosphorylation. Therefore, PRNCR1/miR-377/CCND2 axis repressed cell apoptosis while enhanced cell proliferation in breast cancer via accelerating the MEK/MAPK pathway [164]. Around 70–80% of breast cancer cases are ER+ that plays a vital role in tumor growth and patients survival [165]. Therefore, adjuvant targeted therapies with tamoxifen or aromatase inhibitors are the primary treatment options for ER+ BC patients [166]. However, resistance to hormonal therapy can occur due to the development of estrogen-independent growth. Linc-RoR induced tamoxifen resistance and estrogen-independent growth in BC cells. It activated the MAPK/ERK signaling axis via destabilizing DUSP7 as a suppressor of ERK, indicating the regulatory function of linc-RoR on the MAPK/ERK axis under estrogen deprivation [167]. MiRNA-host gene lncRNAs (lnc-miRHGs) are certain lncRNAs that contain miRNAs within their DNA sequences [168]. OTX1, as a homeobox gene is a pivotal regulator of early human fetal retina and mammary gland development [169]. ERK/MAPK signaling pathway plays a critical role in tumor cell growth, differentiation, angiogenesis, and metastasis [170, 171]. There was MIR100HG upregulation in TNBC tissues and cell lines that increased proliferation, invasion, and migration via targeting the miR-5590-3p/OTX1 axis. MIR100HG inhibition also repressed the ERK/MAPK pathway in TNBC cells [172].

The premetastatic niche (PMN) of primary tumors is a pivotal factor for the metastasis and colonization of tumor cells in certain secondary tissues via organotropism, immunosuppression, angiogenesis, and vascular permeability [173]. CAFs as the principal stromal constituents of the tumor microenvironment regulate tumor progression [174]. CAFs induce tumor proliferation and metastasis via metabolic and extracellular remodeling, cytokines, and exosomes in the primary tumor microenvironment [175–179]. CAFs are pivotal modulators of angiogenesis by WNT5a, WNT2, SDF1, PDGFC, and VEGFA release [180–183]. There was significant SNHG5 up regulation in primary breast CAFs that modulated generation of PMN through angiogenesis. The interaction of SNHG5 and IGF2BP2 stabilized the ZNF281 mRNA in an m6A-dependent manner. ZNF281 modulated CCL2 and CCL5 expressions in CAFs, which resulted in p38 signaling activation in endothelial cells and subsequent PMN construction in the metastatic environment. Recruitment of IGF2BP2 by SNHG5 elevated the ZNF281 levels via m6A regulation, which induced CCL2/5 transcription and secretion to form PMN. CCL2 and CCL5 that were released by CAFs stimulated the p38 MAPK axis in endothelial cells, thereby regulating PMN generation [184].

## Conclusions

Cytokine and growth factor-dependent signaling pathways play a key role during BC progression. In addition to growth factors and cytokines, lncRNAs have also pivotal roles in regulation of these signaling pathways. Therefore, here we discussed the role of lncRNAs in regulation of PI3K/AKT, MAPK, and TGF-β signaling pathways in breast tumor cells. It has been reported that lncRNAs mainly have an oncogenic role through the activation of these signaling pathways during BC progression. Therefore, the inhibition of lncRNAs can be introduced as a suitable therapeutic strategy to reduce the breast tumor growth by suppression of PI3K/AKT, MAPK, and TGF-β signaling pathways. Regarding, the tissue-specific characteristics of lncRNAs, they can also be suggested as the next generation tumor markers. Considering the up regulation of the majority of lncRNAs in tumor tissue and serum of BC patients, lncRNAs expression profiling can be introduced as an efficient non-invasive diagnostic method among these patients. However, regarding the role of lncRNAs in chronic disorders such as diabetes and metabolic disorders, it is required to assess the circulating levels of various lncRNAs in breast cancer patients to introduce a multi lncRNA panel marker instead of a single lncRNA as a non-invasive diagnostic method in these patients. While, the oncogenic lncRNAs can be inactivated using the antisense methods, tumor suppressive lncRNAs can be synthetically-engineered and employed to inhibit breast tumor growth. However, as the breast cancer is a heterogenic malignancy, the personalized medicine is required to use the lncRNAs as the therapeutic factors in breast cancer patients. Therefore, more clinical trials and in-vivo studies are required to bring the lncRNAs as the diagnostic and therapeutic options into the clinics for the breast cancer patients.

## Data Availability

The datasets used and/or analyzed during the current study are available from the corresponding author on reasonable request.

## Abbreviations

ANXA1: Annexin A1
BC: Breast cancer
CAFs: Cancer-associated fibroblasts
DOX: Doxorubicin
EZH2: Enhancer of zeste homolog 2
HER-2: Epidermal growth factor receptor 2
EMT: Epithelial-to-mesenchymal transition
ER: Estrogen receptor
GPCRs: G-protein-coupled receptors
HR: Hormone receptor
LAMC1: Laminin subunit gamma 1
lncRNAs: Long non-coding RNAs
ncRNA: Non-coding RNAs
PRC2: Polycomb Repressive Complex 2
PMN: Premetastatic niche
PR: Progesterone receptor
PUM2: Pumilio RNA binding family member 2
RTKs: Receptor tyrosine kinases
RBPs: RNA-binding proteins
RUNX2: Runt-related transcription factor 2
TAM: Tamoxifen
TGF-β: Transforming growth factor β
TNBC: Triple negative breast cancer
